# Vicus: Exploiting local structures to improve network-based analysis of biological data

**DOI:** 10.1371/journal.pcbi.1005621

**Published:** 2017-10-12

**Authors:** Bo Wang, Lin Huang, Yuke Zhu, Anshul Kundaje, Serafim Batzoglou, Anna Goldenberg

**Affiliations:** 1 Department of Computer Science, Stanford University, Stanford, California, United States of America; 2 Genetics Department, Stanford University, Stanford, California, United States of America; 3 SickKids Research Institute, Toronto, Ontario, Canada; 4 Department of Computer Science, University of Toronto, Toronto, Ontario, Canada; University of California, San Diego, UNITED STATES

## Abstract

Biological networks entail important topological features and patterns critical to understanding interactions within complicated biological systems. Despite a great progress in understanding their structure, much more can be done to improve our inference and network analysis. Spectral methods play a key role in many network-based applications. Fundamental to spectral methods is the Laplacian, a matrix that captures the global structure of the network. Unfortunately, the Laplacian does not take into account intricacies of the network’s local structure and is sensitive to noise in the network. These two properties are fundamental to biological networks and cannot be ignored. We propose an alternative matrix *Vicus*. The Vicus matrix captures the local neighborhood structure of the network and thus is more effective at modeling biological interactions. We demonstrate the advantages of Vicus in the context of spectral methods by extensive empirical benchmarking on tasks such as single cell dimensionality reduction, protein module discovery and ranking genes for cancer subtyping. Our experiments show that using Vicus, spectral methods result in more accurate and robust performance in all of these tasks.

## Author summary

Networks are a representation of choice for many problems in biology and medicine including protein interactions, metabolic pathways, evolutionary biology, cancer subtyping and disease modeling to name a few. The key to much of network analysis lies in the spectrum decomposition represented by eigenvectors of the network Laplacian. While possessing many desirable algebraic properties, Laplacian lacks the power to capture fine-grained structure of the underlying network. Our novel matrix, Vicus, introduced in this work, takes advantage of the local structure of the network while preserving algebraic properties of the Laplacian. We show that using Vicus in spectral methods leads to superior performance across fundamental biological tasks such as dimensionality reduction in single cell analysis, identifying genes for cancer subtyping and identifying protein modules in a PPI network. We postulate, that in tasks where it is important to take into account local network information, spectral-based methods should be using Vicus matrix in place of Laplacian.

This is a *PLOS Computational Biology* Methods paper.

## Introduction

Networks are a powerful paradigm for representing relations among objects from micro to macro level. It is no surprise that networks became a representation of choice for many problems in biology and medicine including gene-gene and protein-protein interaction networks [1], diseases [2] and their interrelations [3], cancer subtyping [4], genetic diversity [5], image retrieval [6], dimensionality reduction [7, 8] and many other applications. Computational biologists routinely use networks to represent data and analyze networks to obtain better understanding of patterns and local structures hidden in the complex data they encode. One of the most standard graph-based methods to analyze networks is to decompose it into eigenvectors and eigenvalues, i.e. apply spectral methods to the network to understand its structure. At the heart of spectral methods is the so-called Laplacian matrix. Spectral clustering relies on the fact that the principle eigenvectors of the Laplacian capture membership of nodes in implicit network clusters. This principle is essential to clustering and dimensionality reduction.

The traditional formulation of the Laplacian captures the global structure of the matrix, which is often insufficient in biology where local topologies are what needs to be sought and exploited. Moreover, recently algorithms designed to capture the local structure of the data have been shown to significantly outperform global methods [9, 10]. These approaches aim to reconstruct each data point using its local neighbours and have been shown to be robust and powerful for unweighted networks. Weighted networks are richer representations of underlying data than unweighted networks: in biological networks weights can represent the strength of interactions or the strength of the evidence underlying each interaction, in patient networks weights represent the degree of similarity between patients [4]. In this paper, we provide a local formulation of the Laplacian for weighted networks which we call the *Vicus* matrix ($V^{+}$), from the Latin word ‘neighborhood’. Using Vicus in place of the Laplacian allows spectral methods to exploit local structures and makes them a lot more relevant to a variety of biological applications.

In this paper we introduce Vicus and compare its performance to the Laplacian across a wide range of tasks. Our experiments include single cell dimensionality reduction, protein module discovery, feature ranking and large scale network clustering. Since we consider such a diverse set of biological questions, in each case we also compare to appropriate state-of-the-art methods corresponding to each question. Spectral clustering using Vicus outperforms competing approaches in all of these tasks. Our experiments show that Vicus is a more robust alternative to traditional Laplacian matrix for network analysis.

## Results

### Simulations: Laplacian vs Vicus

In this section we consider predetermined 2D and 3D structures, represent them as a graph and analyze the performance of local Vicus as compared to traditional Laplacian in the task of graph-based dimensionality reduction.

**Fig 1:**
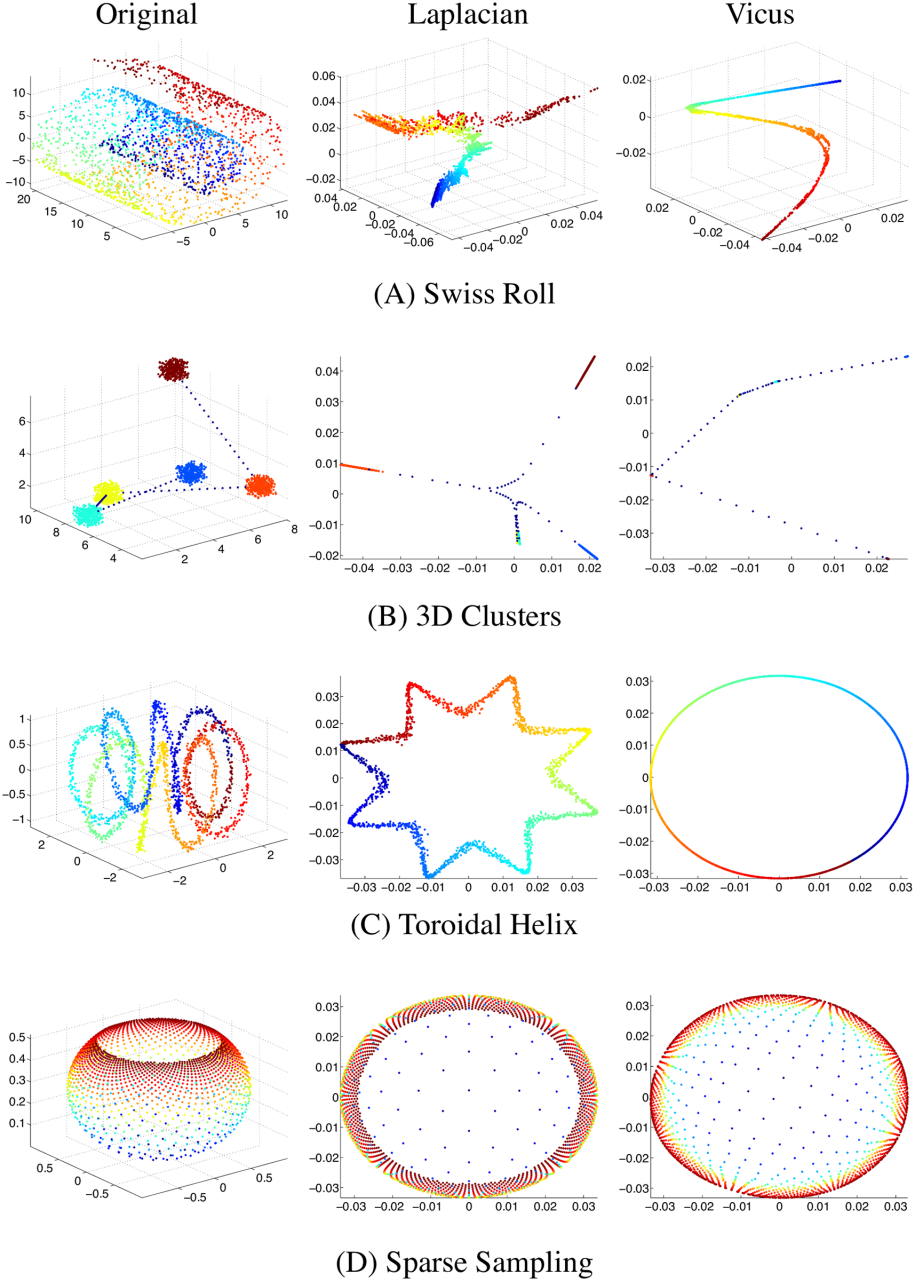
Four examples of three dimensional manifolds (left column) and their embeddings by global Laplacian (middle column) and the proposed Vicus (right column). A shows an example of the structure known as jelly roll or Swiss roll which is particularly common in viral proteins. B shows five random non-overlapping clusters in 3D space connected by sparsely measured channels. C shows a toroidal helix containing a circle as its basic geometric shape. D is an example of sampling in 3D space where we sample points from a solid bowl-shaped figure non-uniformly: the top of the bowl is more densely sampled, gradually reducing sampling towards the bottom of the bowl graph. On all the cases, Vicus is able to recover the underlying distributions of the input data more robustly.

First, let us consider a particular type of protein fold that has a complex structure in which four pairs of antiparallel beta sheets, only one of which is adjacent in sequence, are wrapped in three dimensions to form a barrel shape. This structure known as jelly roll or Swiss roll is particularly common in viral proteins and is schematically depicted in Fig 1A. Spectral methods assume that clusters of data points can be well described by the Euclidean distance. Though it looks relatively unambiguous to a human, this task is computationally challenging since the assumption that Euclidean proximity translates to similarity does not hold in the original data space for the Swiss roll structure. As expected, standard spectral decomposition fails to find a lower dimensional representation of the data due to the inability to capture the underlying manifolds in Fig 1A. Using Vicus in place of the Laplacian matrix helps spectral decomposition to transform the original data to the latent space with reduced complexity while preserving the contiguity and the cluster memberships of the original data.

Another simulation that we considered is a typical example in bioinformatic imaging, structured 3D data. A schematic of clustered signal within brain regions and connecting channels between them is captured in Fig 1B. Given five random non-overlapping clusters in 3D space connected by sparsely measured channels, Vicus maps the clusters into dense points while preserving the lines connecting them. This embedding indicates that, by considering local structures, local spectrum can highlight the obvious cluster structures without disregarding the structure of the data between clusters. By comparison, Laplacian-based embedding highlights the dense clusters while making the connectivity between them more ambiguous (Fig 1B). This example sheds light on how Vicus can preserve local structure of the data.

A very common structure in protein folding is a helix. Among such foldings are toroidal helices, where the helix is wrapped around a toroid. These structures have a pore in the middle that allows unfolded DNA to pass through. The toroidal helix in Fig 1C has a circle as its basic geometric shape. Our local spectrum recovers the underlying 2D circle by considering the labels in local neighborhoods while the Laplacian finds a circle distorted by similarities of points in the 3D dimension. The distortions by the global spectrum result from a fundamental limitation in descriptive power of Euclidean distance in high dimensional spaces, while our local spectrum can avoid such limitation by focusing on the local rather than the global manifold structures.

Our final example is the task of sampling in 3D space, such as sampling an image of a cell shape in a cell morphology study. We sampled points from a solid bowl-shaped figure (Fig 1D) non-uniformly: the top of the bowl is more densely sampled, gradually reducing sampling towards the bottom of the bowl graph. The Laplacian based 2D embedding has considerable bias towards the densely sampled region while Vicus’ embedding recognizes that sampling was done on a solid shape, again by capturing the labels in the local neighbourhoods.

These examples show the benefits of capturing local structure in a network (graph) decomposition, which gives a better understanding of patterns and neighborhoods hidden in complex networks.

### A case study in single-cell RNA-seq analysis

Single-cell RNA sequencing (scRNA-seq) technologies have recently emerged as a powerful means to measure gene expression levels of individual cells [11]. Quantifying the variation across gene expression profiles of individual cells is key to the dissection of the heterogeneity and the identification of new populations among cells. The unique challenges associated with single-cell RNA-seq data include large noise in quantification of transcriptomes and high dropout rates, therefore reducing the usability of traditional unsupervised clustering methods. Vicus, employing local structures hidden in high-dimensional data, is able to tackle these challenges and improve many types of single-cell analyses including visualization, clustering and gene selection.

We benchmark our method on four recently published single-cell RNA-seq datasets with validated cell populations:

- Pollen data set [12] consists of 11 cell populations including neural cells and blood cells.
- Usoskin data set [13] consists of neuronal cells with sensory subtypes. This data set contains 622 cells from the mouse dorsal root ganglion, with an average of 1.14 million reads per cell. The authors divided the cells into four neuronal types: peptidergic nociceptors, non-peptidergic nociceptors, containing neurofilament and containing tyrosine hydroxylase.
- Buettner data set [14] consists of embryonic stem cells in different cell cycle stages. This dataset was obtained from a controlled study that quantified the effect of the cell cycle on gene expression level in individual mouse embryonic stem cells (mESCs).
- Kolodziejczyk data set [15] consists of pluripotent cells under different environmental conditions. This data set was obtained from a stem cell study on how different culture conditions influence pluripotent states of mESCs. Preprocessed data was obtained directly from [11].

The main reason we chose these four single-cell datasets is that their ground-truth labels have been validated either experimentally or computationally in their original studies. We formulate the problem of clustering cells from RNA-seq data in terms of networks. First, cell-to-cell similarity networks (Materials and methods) are constructed from single-cell RNA-seq data. The advantage of using networks to represent this data are in network’s ability to capture a set of relationship between all pairs of cells. After the construction of cell-to-cell networks, we can apply our Vicus to obtain a low-dimensional representation that contains local structures in the networks and potential cluster memberships of cells.

**Fig 2:**
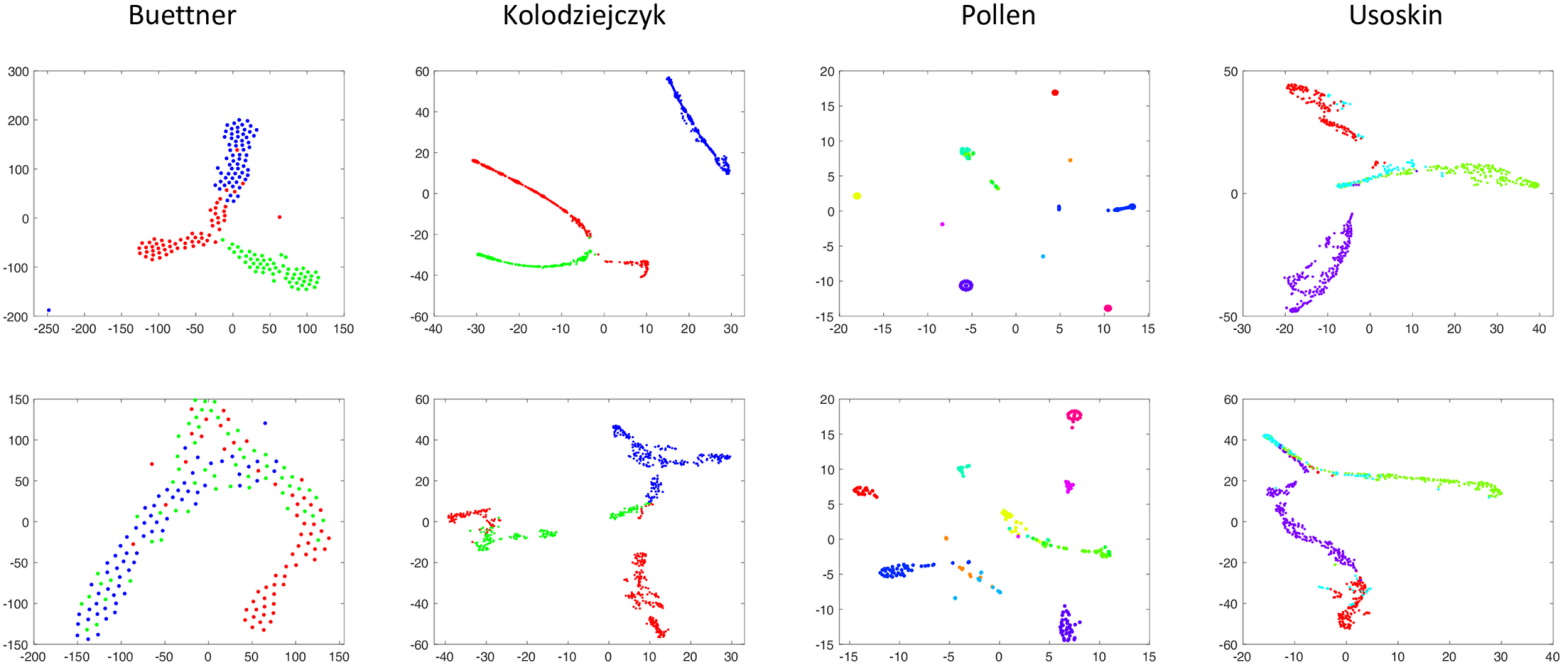
Visualization of low-dimensional representations for single cells learned by Vicus and global Laplacian. Four columns represent the embedding results for Buettner data, Kolodziejczyk data, Pollen data, and Usoskin data respectively. In each dataset, cells are color-coded as their ground-truth labels. Larger separations between different clusters usually indicate better performances in low-dimensional embeddings.

To demonstrate the representative power of the low-dimensional representations by Vicus, we ran t-SNE [16], the most common visualization method in single-cell studies, on the obtained low-dimensional representations and compare the 2-D visualization of both Vicus and Laplacian across the four single-cell datasets in Fig 2. Note that we are only using t-SNE for the purpose of visualization of Laplacian and Vicus. The cells, color-coded by the ground-truth labels from original studies [12–15], are clearly separated by Vicus (Fig 2), indicating greater power of Vicus to capture fine-grained structures in cell-to-cell similarity networks.

We compare spectral decomposition using Vicus with spectral methods using traditional global Laplacian along with 6 other popular dimensionality reduction methods. The six methods include linear methods such as Principle Component Analysis (PCA), Factor Analysis(FA), and Probabilistic PCA (PPCA) and nonlinear methods such as multidimensional scaling (MDS), Kernel PCA, Maximum Variance Unfolding (MVU), Locality Preserving Projection (LPP) and Sammon mapping. We use a widely-used toolbox [16] implementing all these popular dimensionality reduction methods. Further, we also compare Vicus with three widely used state-of-the-art network-based clustering algorithms: InfoMap [17], modularity-based Louvian [18], and Affinity Propagation (AP) [19]. To compare these 11 methods we adopted two metrics: Normalized Mutual Information(NMI) [20] and Adjusted Rand Index(ARI) [21] (Materials and methods), evaluating the concordance of obtained label and the ground-truth. Higher values of these evaluation metrics indicate better ability of correctly identifying cell populations.

Results in Table 1 illustrate Vicus’ superior performances compared to all ten other methods in most of the considered cases. It is noticeable that Vicus outputs much better module detection results than all other methods on Buettner data set [14]. This is due to the fact that Buettner data set [14] contains cells in three different continuous cell stages which are hard to detect due to large noise. In addition, PCA performs the best on Pollen data set [12] because the ground-truth is obtained by simple clustering with PCA on a set of pre-selected genes. Further, compared with the three network-based module detection methods (InfoMap, Louvian and AP), our Vicus is able to achieve much more accurate module discovery on each of the same networks.

**Table 1:** Clustering results comparison on the four single-cell datasets.

*NMI*/*ARI*	Buettner	Kolodziejczk	Pollen	Usoskin
PCA	0.429/0.394	0.553/0.539	0.946/0.941	0.468/0.395
FA	0.337/0.278	0.686/0.679	0.700/0.558	0.135/0.104
PPCA	0.182/0.174	0.770/0.727	0.922/0.890	0.694/0.731
MDS	0.429/0.395	0.557/0.543	0.931/0.885	0.468/0.438
Sammon	0.247/0.232	0.434/0.423	0.903/0.825	0.582/0.551
KPCA	0.286/0.204	0.413/0.339	0.701/0.692	0.268/0.189
LPP	0.314/0.229	0.720/0.699	0.890/0.801	0.632/0.654
MVU	0.247/0.154	0.610/0.652	0.839/0.690	0.226/0.175
InfoMap	0.584/0.274	0.688/0.443	0.930/0.884	0.580/0.257
Louvian	0.731/0.654	0.728/0.599	0.770/0.643	0.603/0.561
AP	0.214/0.135	0.712/0.699	0.816/0.671	0.256/0.172
Global Laplacian	0.271/0.166	0.600/0.495	0.855/0.790	0.592/0.555
Vicus	0.778/0.742	0.780/0.719	0.934/0.880	0.695/0.701

### Vicus captures rare cell populations

**Fig 3:**
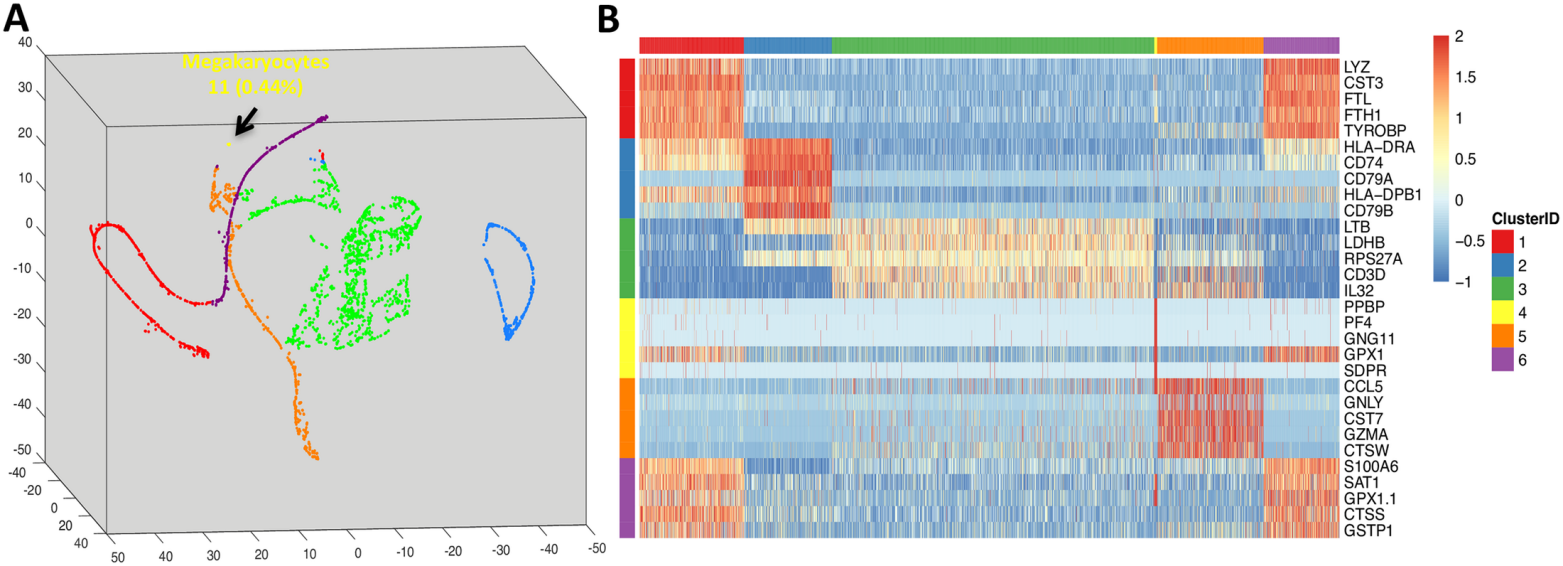
Vicus detects rare population in PBMC data. A: a 3-D mapping of the learned low dimension by Vicus. Each cell is colored according to its ground-truth. The rare population of Megakaryocytes is shown in yellow. B: The top 5 differential genes for each cell types detected by Vicus.

One of the major challenges in single-cell analysis is to detect rare populations of cells from noisy single-cell RNA-seq data. The signals of rare populations can be easily neglected due to the existence of various sources of noises. Our approach based on Vicus matrix is able to discover weak signals of rare populations by exploiting local structures while global Laplacian fails. We applied our method on a scRNA-seq data consisting of 2700 peripheral blood mononuclear cells (PBMC). It is generated by 10x Genomics GemCode platform, a droplet-based high-throughput technique and 2700 cells with UMI counts were identified by their customized computational pipeline [22]. This cell population includes five major immune cell types in a healthy human as well as a rare population of metakaryocytes (less than 0.5% abundance in PBMC). The processed data is available in [11] and was originally published in [22]. Vicus captures the rare population consisting of 11 cells (Fig 3A) while global Laplacian fails to find such rare population. Vicus is also able to detect differential genes that define each cluster (Fig 3B). Note that we used Vicus score to rank important genes (Materials and methods) and we only show top 5 genes for each cluster.

### Stability in clustering *E. coli* PPI network

Identification of functional modules in Protein-protein interaction (PPI) networks is an important challenge in bioinformatics. Network module detection algorithms can be employed to extract functionally homogenous proteins. In this application, first submodules are detected and subsequently these submodules are investigated for enrichment of proteins with a particular biological function. Stability is one of the essential goals of the multi-scale module detection problem [23]. It measures how robust the employed algorithm is able to recover the most dense subnetworks enriched to certain biological functions or physical interactions. Inside the definition of stability (Materials and methods), the Laplacian is used in a Markov process on the network which allows to compare and rank partitions at each iteration.

**Fig 4:**
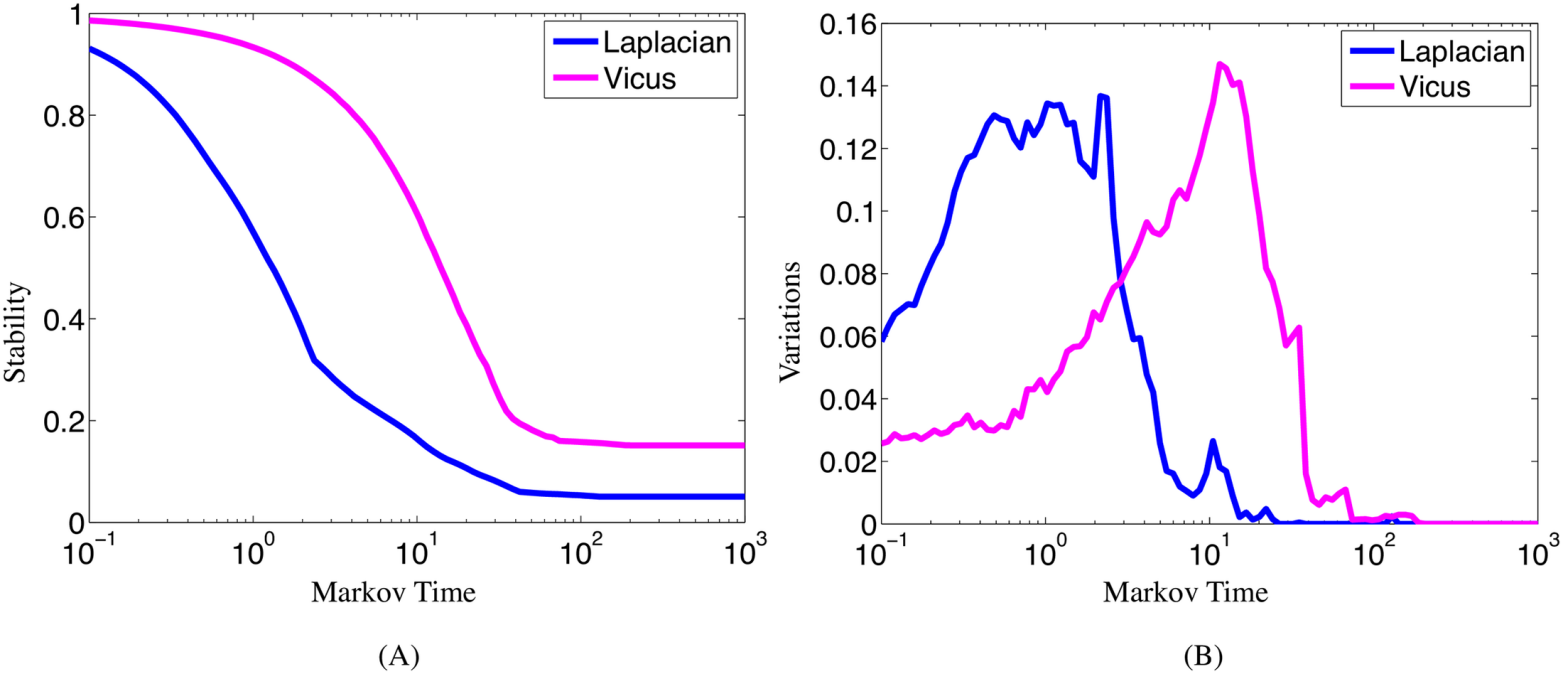
Stability and variations along different Markov time spans. Stability in Panel A indicates the robustness of the community detection algorithms (Vicus vs Laplacian) while Variations in Panel B show how the corresponding algorithm exploits the community membership information in the network.

To analyze the stability of our method we partition a Protein-Protein Interaction(PPI) network, which consists of 7,613 interactions between 2,283 Escherichia coli proteins [24]. This task is more challenging than traditional clustering problems due to the intrinsic complexity of the cell captured by the PPI network. Due to large noise in experimental measurements of protein interactions, proteins in the same pathway do not necessarily have higher density of interactions. This fact poses particular challenges to traditional network partition algorithms which usually fail to infer the true membership of proteins to their underlying pathways. Vicus-based spectrum exhibits higher stability along the Markovian timeline (Fig 4A) compared to the global Laplacian. Global spectrum and Vicus-based local spectrum exploit different modes of variation in the network (Fig 4B). Global spectrum tends to find large components in networks to reduce the variation and increase stability while local spectrum exploits deeper substructures of the large components and detects partitions in more fine-grained fashion.

### Ranking genes associated with cancer subtypes

One of the holy grails of computational medicine is identification of robust biomarkers associated with the phenotype of interest. Here we consider the question of identifying genes associated with cancer subtyping in 5 cancers from 6 microarray datasets. These are benchmark datasets for feature selection in computational biology from http://featureselection.asu.edu/datasets.php. Table 2 shows the statistics of these six datasets.

**Table 2:** Statistics summary of the six macro-array datasets for cancer subtypes.

Data Set	# Instances	# Features	# Classes	Attributes
ALLAML	72	7129	2	continuous, binary
Carcinom	174	9182	11	continuous, multi-class
GLIOMA	50	4434	4	continuous, multi-class
leukemia	72	7070	2	discrete, binary
lung	203	3312	5	continuous, multi-class
lung-discrete	73	325	7	discrete, binary

In the standard formulation of spectral clustering, the ranking of features (in this case, genes) is done using Laplacian score. Laplacian Score is a score derived based on the network spectrum that is commonly used to rank features in the order of their importance and relevance to the clusters. Given a feature **f**, the corresponding Laplacian Score (LS) is defined as follows:

$$\begin{array}{c} LS\left(f\right)=\frac{f^{T}L^{+}f}{f^{T}f}. \end{array}$$ (1)

Unfortunately, LS has difficulty identifying features that are only relevant to one of the clusters (a certain local subnetwork) but not the whole network. Traditional LS will prefer features that are globally relevant to all the clusters, even if they are not as strongly indicative of any cluster in particular. We thus, propose to substitute the Laplacian matrix $L^{+}$ with our Vicus matrix $V^{+}$. We define our Vicus Score (VS) analogously to Laplacian Score:

$$\begin{array}{c} VS\left(f\right)=\frac{f^{T}V^{+}f}{f^{T}f}. \end{array}$$ (2)

**Fig 5:**
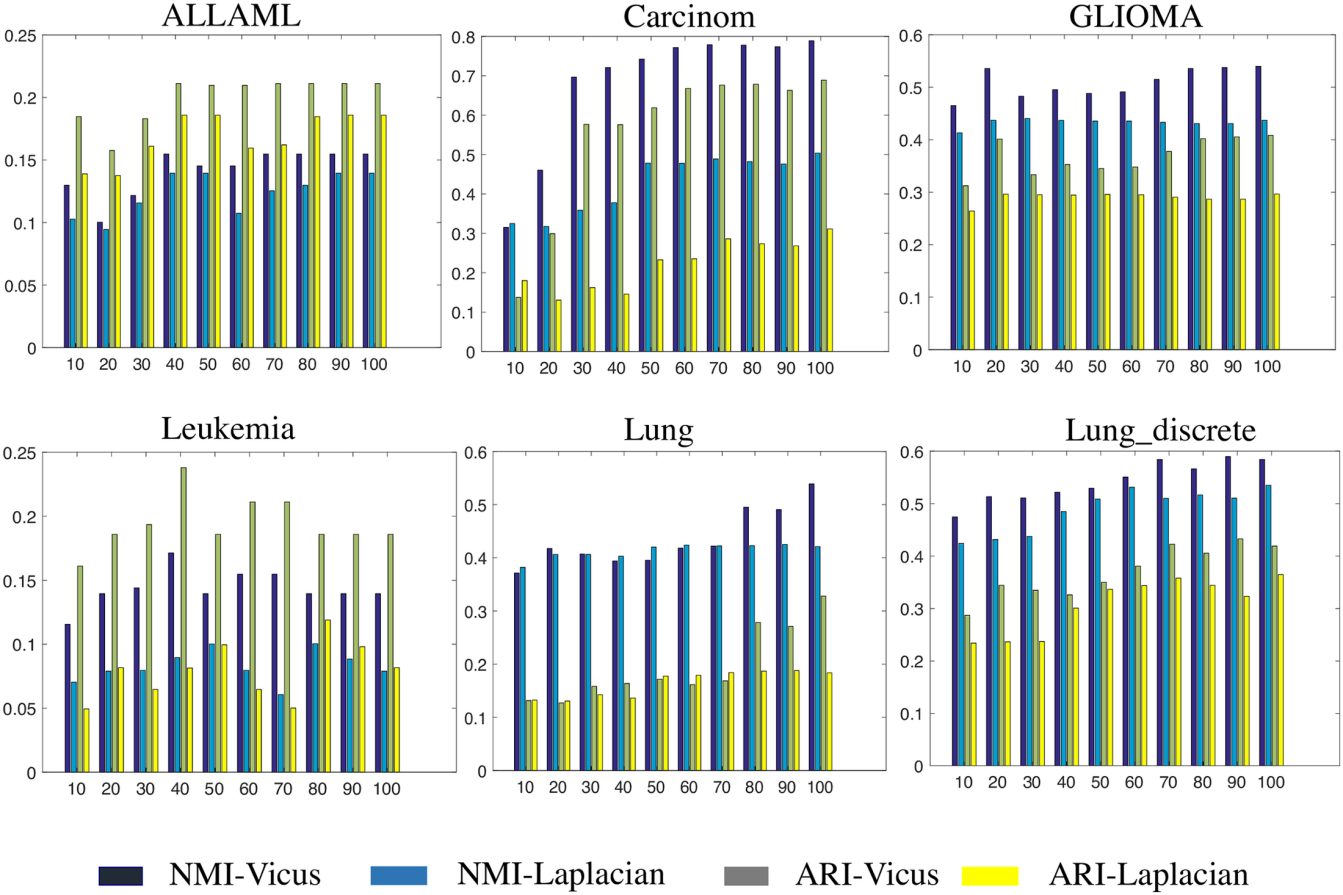
The results of feature ranking by Laplacian and Vicus. Experiments are performed on 6 cancer datasets. On each dataset, we vary the number of selected features (genes) and use k-means to report the clustering accuracy. NMI and ARI are used to measure the goodness of selected features. It is consistently observed across six datasets that Vicus can select better features than Laplacian.

For each data set presented in Table 2, we rank the features by Laplacian Score and Vicus Score. We take *N* highest ranked features and then apply simple k-means clustering. If the feature ranking algorithm correctly ranks the relevant features, the clustering accuracy should be higher compared to the accuracy of the method that uses the same number of chosen but less relevant features. We varied the number of chosen features and plotted the accuracy of the ranking algorithms in Fig 5. Again, we use NMI and ARI as the evaluation metrics for the clustering results. We observe that features ranked using the Vicus matrix result in better accuracy when the number of chosen features is small, confirming that the most discriminative features are ranked among the top by Vicus.

## Discussion

The proposed Vicus matrix for weighted networks exhibits greater power to represent the underlying cluster structures of the networks than the traditional global Laplacian. The key observation is the ability of our local spectrum to make the top eigenvectors more robust to noise and hyper parameters in the process of constructing such weighted networks. The proposed Vicus-based local spectrum can supplant the usage of Laplacian-based spectral methods for weighted networks in various tasks such as clustering, community detection, feature ranking and dimensionality reduction. Sharing similar algebraic properties with global Laplacian, our local spectrum helps to understand the underlying structures of the noisy weighted networks. As demonstrated, local spectrum is robust with respect to noise and outliers. Finally, we have parallelized Vicus to achieve scalability. While the discussed applications contained at most a few thousands nodes, we have performed experiments on networks with up to 500,000 nodes. On this very large network, Laplacian based spectral clustering took 7.5min while Vicus took 12.9min with better performance (higher NMI). Thus, Vicus is not only more accurate but it can scale to very large networks, a property which will become important as we start constructing, for example, DNA co-methylation probe-based networks with hundreds of thousands of probes.

### Conclusion

The power of local network neighborhoods has become abundantly clear in many fields where the networks are used. Principled methods are needed to take advantage of the local network structure. In this work we have proposed the Vicus matrix, a new formulation that shares algebraic properties with the traditional Laplacian and yet improves the power of spectral methods across a wide range of tasks necessary to gain deeper understanding into biological data and behavior of the cell. Taking advantage of the local network structure, we showed improved performance in single cell RNA-seq clustering, feature ranking for identifying biomarkers associated with cancer subtyping and dimensionality reduction in single cell RNA-seq data. Further, we have shown that our method is amenable to parallelization which allows it to be performed in time comparable to the traditional methods.

## Materials and methods

### Laplacian matrix

Suppose we have a network G={V,E} with a set of *V* nodes and E weighted edges. Let $W\in R^{\left|V|\times |V\right|}$ be the weighted ajacency matrix of this network, where |*V*| is the number of nodes. Here, *W*_*ij*_ represents the weight of the edge between the *i*th and *j*th nodes. Let diagonal matrix *D* be *W*’s degree matrix, where $D_{ii}=\sum_{j}^{\left|V\right|}W_{ij}$. The classical formulation of the Laplacian of *W* is then matrix L=D-W also known as the *combinatorial Laplacian*. A common variant of the Laplacian L is $L^{+}=I-D^{-1/2}WD^{-1/2}$ which is called the *normalized Laplacian*.

Traditional state-of-the-art spectral clustering [25] aims to minimize RatioCut, an objective function that effectively combines MinCut and equipartitioning, by solving the following optimization problem:

$$\begin{array}{ccc} \min_{Q\in R^{n\times C}} & & Trace(Q^{T}L^{+}Q)\\ s.t. & & Q^{T}Q=I. \end{array}$$ (3)

where *C* is the number of clusters, *n* is the number of nodes and **Q** = [**q**^1^, **q**^2^, …, **q**^*C*^] is the set of eigenvectors, capturing the structure of the graph. Eigenvectors associated with the Laplacian matrix of the weighted network are used in many tasks (e.g., face clustering, dimensionality reduction, image retrieval, feature ranking, etc). These eigenvectors suffer from some limitations. For example, the top eigenvectors, in spite of their ability to map the data to a low-dimensional space, are sensitive to noisy measurements and outliers encoded by pairwise similarities (S1 Fig) [4]. Additionally, the Laplacian is very sensitive to the hyper-parameters used to construct the similarity matrices (Materials and methods, S2 Fig) [25].

### Local spectrum matrix: *Vicus*

Our Vicus Matrix ($V^{+}$) is similar to the Laplacian ($L^{+}$) in functionality and in addition captures the local structure inherent in the data. The intuition behind Vicus is that we use local information from neighboring nodes, akin to label propagation [26] or random walks [27]. As we demonstrate, relying on local subnetworks makes the matrix more robust to noise, helping to alleviate the influence of outliers.

Let our data be a set of points {*x*_1_, *x*_2_, …, *x*_*n*_}. Then, each vertex *v*_*i*_, in the weighted network G, represents a point *x*_*i*_ and $N_{i}$ represents *x*_*i*_’s neighbours, *not* including *x*_*i*_. We constrain the neighbourhood size to be held constant across nodes (i.e., $∥N_{i}∥=K,i=1,2,\ldots ,n$).

Our main assumption is that the labels (such as cluster assignments 1 … *C* for *C* clusters) of neighbouring points in the network are similar. Specifically, we assume that the cluster indicator value of the *i*^*th*^ datapoint (*x*_*i*_) can be inferred from the labels of its direct neighbors ($N_{i}$). First, we extract a subnetwork $G_{i}=\left(V_{i},E_{i}\right)$ such that $V_{i}=N_{i}\cup x_{i}$ and $E_{i}=E\left(V_{i}\right)$ which represents the edges connecting all the nodes in *V*_*i*_. The similarity matrix associated with the subgraph $G_{i}$ is $W^{i}=W\left(E_{i}\right)$, representing the weights for all the edges associated with all the nodes in *V*_*i*_. Using the label diffusion algorithm [28], we can reconstruct a virtual label indicator vector $p_{V_{i}}^{k}$ such that

$$\begin{array}{c} p_{V_{i}}^{k}=\left(1-\alpha \right){\left(I-\alpha S_{i}\right)}^{-1}q_{V_{i}}^{k},1\leq k\leq C, \end{array}$$ (4)

where *α* is a constant (0 < *α* < 1, empirically set to 0.9 in all our experiments, as suggested in [28]) and $q_{V_{i}}^{k}$ is the scaled cluster indicator vector of the subnetwork $G_{i}$. **S**_*i*_ represents the normalized transition matrix of *W*^*i*^, i.e., $S_{i}\left(u,t\right)=\frac{W^{i}\left(u,t\right)}{\sum_{l=1}^{K+1}W^{i}\left(u,l\right)}$. Note that we do not actually perform any diffusion, since our setting is completely unsupervised. Instead we use **p**_*k*_ to estimate *q*_*ik*_. $p_{V_{i}}^{k}$ is a vector of *K* + 1 elements, where ${\hat{q}}_{i}^{k}=p_{V_{i}}^{k}\left[K+1\right]$ is the estimate of how likely datapoint *i* belongs to cluster *k* based on its neighbours. As we want maximal concordance between ${\hat{q}}_{i}^{k}$ and $q_{i}^{k}$, we set ${\hat{q}}_{i}^{k}=\beta_{i}q_{V_{i}}^{k}$, where $\beta_{i}\in R^{K+1}$ is the row of the matrix (1 − *α*)(*I* − *α***S**_*i*_)^−1^, representing label propagation at its final state. Here, *β*_*i*_ represents the convergence of the label propagation for the datapoint *i* (Note that the original matrix was constructed as the concatenation of the neighborhood of *i* and datapoint *i* as the last row). Hence

$$\begin{array}{c} {\hat{q}}_{i}^{k}\approx \frac{\beta_{i}\left[1:K\right]q_{N_{i}}^{k}}{1-\beta_{i}\left[K+1\right]}; \end{array}$$ (5)

where *β*_*i*_[1: *K*] represents the first *K* elements of *β*_*i*_ and *β*_*i*_[*K* + 1] is the *K* + 1st element in *β*_*i*_, corresponding to the *i*^*th*^ datapoint.

We can construct a matrix *B*, that represents a linear relationship ${\hat{q}}^{k}\approx Bq^{k}$, (*k* = 1, …, *C*), such that

$$\begin{array}{c} B_{ij}=\{\begin{array}{cc} \frac{\beta_{i}\left[j\right]}{1-\beta_{i}\left[K+1\right]} & \;\text{if}\;x_{j}\in N_{i}\;\text{and}\;x_{j}\;\text{is}\;\text{the}\;j\text{-th}\;\text{element}\;\text{in}\;N_{i}\\ 0 & \;\text{otherwise} \end{array} \end{array}$$ (6)

Our objective is to minimize the difference between ${\hat{q}}^{k}$ and **q**^*k*^:

$$\begin{array}{ccc} \sum_{i=1}^{n}\sum_{k=1}^{C}{\left({\hat{q}}_{i}^{k}-q_{i}^{k}\right)}^{2}\; & = & \sum_{k=1}^{C}{∥q^{k}-{\hat{q}}^{k}∥}^{2}\approx \sum_{k=1}^{C}{∥q^{k}-Bq^{k}∥}^{2}\\ \;\; & = & Trace(Q^{T}{\left(I-B\right)}^{T}\left(I-B\right)Q) \end{array}$$ (7)

Setting $V^{+}={\left(I-B\right)}^{T}\left(I-B\right)$, we arrive at our novel *local* version of spectral clustering:

$$\begin{array}{ccc} \min_{Q\in R^{n\times C}} & & Trace(Q^{T}V^{+}Q)\\ s.t. & & Q^{T}Q=I. \end{array}$$ (8)

Similarly to the original spectral clustering formulation (Eq 3), our clustering results can be obtained by performing eigen-decomposition of matrix $V^{+}$ [25] to solve Eq 8. The final grouping of datapoints into clusters is achieved by performing k-means clustering on **Q** as in [29].

#### Analysis of Vicus properties

Our Vicus Matrix $V^{+}$ shares many properties with Laplacian $L^{+}$ [25]:

1. Both matrices are symmetric and positive semi-definite.
2. The smallest eigenvalue of both matrices is 0, the corresponding eigenvector is the constant vector **l**.
3. Both matrices have *n* non-negative, real-valued eigenvalues 0 = *λ*_1_ ≤ *λ*_2_ ≤ … ≤ *λ*_*n*_
4. The multiplicity of the eigenvalue 0 of both $L^{+}$ and $V^{+}$ equals the number of connected components in the network.

Here *n* is the number of nodes in the network. To prove the first property, we note that ${V^{+}}^{T}={\left\{{\left(I-B\right)}^{T}\left(I-B\right)\right\}}^{T}={\left(I-B\right)}^{T}\left(I-B\right)=V^{+}$, thus $V^{+}$ is symmetric. Also, for any non-zero vector **x**, we have $x^{T}*V^{+}*x=x^{T}*{\left(I-B\right)}^{T}\left(I-B\right)*x=||(I-B)*x||\geq 0$.

For the second property, we first prove that *B* **l** = **l**, i.e., matrix *B* has an eigenvalue of 1 corresponding to an all-one constant vector. To prove the above, the following statement must be true:

$$\begin{array}{c} \sum_{j\in N_{i}}B_{ij}=1,i=1,2,\ldots ,n. \end{array}$$

According to Eq 6, $\sum_{j\in N_{i}}B_{ij}=\frac{\sum_{N_{i}}\beta_{i}\left[j\right]}{1-\beta_{i}\left[K+1\right]}$. Note that *β*_*i*_ is the last row of the transition kernel $\left(1-\alpha \right){\left(I-\alpha L_{i}\right)}^{-1}=\left(1-\alpha \right)\sum_{l=0}^{\infty }{\left(\alpha L_{i}\right)}^{l}$, hence we have $\sum_{j\in N_{i}}\beta_{i}\left[j\right]+\beta_{i}\left[K+1\right]=\left(1-\alpha \right)\sum_{l=0}^{\infty }\alpha^{l}=1$, considering the sum of each row of **L**_*i*_ is all one. Thus we prove $\sum_{j\in N_{i}}B_{ij}=1$ and therefore *B***l** = **l**.

It is then easy to verify that

$$\begin{array}{ccc} V^{+}l & = & {\left(I-B\right)}^{T}\left(I-B\right)l\\ & = & (I-B-B^{T}+B^{T}B)l\\ & = & l-l=0\;□ \end{array}$$

Hence we proved that matrix $V^{+}$ always has an eigenvalue of 0 corresponding to the eigenvector **l**. Property 3 follows directly from properties 1 and 2. Finally, the last property can be easily proven using an arguments similar to [25]. The above properties verify that our proposed Vicus matrix is a proper alternative to Laplacian including the desirable algebraic properties.

### Similarity network constructions

Given a feature set that describes a collection of objects, denoted as **X** = {**x**_**1**_, **x**_**2**_, …, **x**_**n**_}, we want to construct a similarity network $N\in R^{n\times n}$ in which N(i,j) indicates the similarity between the *i*-th and *j*-th object. The most widely used method is to assume a Gaussian distributions across pairwise similarites:

$$\begin{array}{c} N\left(i,j\right)=\exp\left(-\frac{∥x_{i}-x_{j}{∥}^{2}}{2\sigma^{2}}\right); \end{array}$$

Here *σ* is a hyper-parameter that needs careful manual setting. More advanced methods of constructing similarity networks can be seen in [4].

### Normalized mutual information

Throughout the paper, we used Normalized Mutual Information (NMI) [20] to evaluate the consistency between the obtained clustering and the groundthuth. Given two clustering results *U* and *V* on a set of data points, NMI is defined as: *I*(*U*, *V*)/ max{*H*(*U*), *H*(*V*)}, where *I*(*U*, *V*) is the mutual information between *U* and *V*, and *H*(*U*) represents the entropy of the clustering *U*. Specifically, assuming that *U* has *P* clusters, and *V* has *Q* clusters, the mutual information is computed as follows:

$$\begin{array}{c} I\left(U,V\right)=\sum_{p=1}^{P}\sum_{q=1}^{Q}\frac{|U_{p}\cap V_{q}|}{N}\log\frac{N|U_{p}\cap V_{q}|}{|U_{p}\left|\times \right|V_{q}|} \end{array}$$

where |*U*_*p*_| and |*V*_*q*_| denote the cardinality of the *p*-th cluster in *U* and the *q*-th cluster in *V* respectively. The entropy of each cluster assignment is calculated by

$$H(U)=-\sum_{p=1}^{P}\frac{\left|U_{p}\right|}{N}\text{log}\frac{\left|U_{p}\right|}{N},$$

and

$$H(V)=-\sum_{q=1}^{Q}\frac{\left|V_{q}\right|}{N}\text{log}\frac{\left|V_{q}\right|}{N}.$$

Details can be found in [20]. NMI is a value between 0 and 1, measuring the concordance of two clustering results. In the simulation, we calculate the obtained clustering with respect to the ground-truth. Therefore, a higher NMI refers to higher concordance with truth, i.e. a more accurate result.

### Adjusted Rand Index

The Adjusted Rand Index (ARI) is another widely-used metric for measuring the concordance between two clustering results. Given two clustering *U* and *V*, we calculate the following four quantities:

*a*: number of objects in a pair are placed in the same group in *U* and in the same group in *V*;
*b*: number of objects in a pair are placed in the same group in *U* and in different groups in *V*;
*c*: number of objects in a pair are placed in the same group in *V* and in different groups in *U*;
*d*: number of objects in a pair are placed in different groups in *U* and in different groups in *V*.

The (normal) Rand Index (RI) is simply $\frac{a+d}{a+b+c+d}$. It basically weights those objects that were classified together and apart in both *U* and *V*. There are some known problems with this simple version of RI such as the fact that the Rand statistic approaches its upper limit of unity as the number of clusters increases. With the intention to overcome these limitations, ARI has been proposed in [21] in the form of

ARI=(n2)(a+d)-[(a+b)(a+c)+(c+d)(b+d)](n2)-[(a+b)(a+c)+(c+d)(b+d)].

### Stability and variations in Markov clustering

Given a network on a set of *N* nodes with edge weights *W*, we first present a few related terms as follows

*L*: the Laplacian matrix for the network. It can either be traditional Laplacian or our newly proposed Vicus;
*π*: the stationary distribution vector with *πL* = 0
*d*_*i*_: the degree of node *i* as *d*_*i*_ = ∑_*j*_ *W*_*ij*_
Σ: the normalized degree matrix with nonzero values only on the diagonal $\Sigma_{ii}=\frac{d_{i}}{\sum_{k}d_{k}}$.
*H*: the partition indicator matrix with *H*_*ij*_ = 1 if the node *i* is classified with cluster *j* and *H*_*ij*_ = 0 otherwise.

Then the stability measure on time *t* is defined in terms of the clustered auto-covariance matrix

$$\begin{array}{c} R_{t}=H^{T}\left(\Sigma {\left(I-L\right)}^{t}-\pi^{T}\pi \right)H \end{array}$$

as follows:

$$\begin{array}{c} r\left(t;H\right)=\min_{0\leq s\leq t}\sum_{i=1}^{C}{\left(R_{s}\right)}_{ii}=\min_{0\leq s\leq t}trace\left(R_{s}\right), \end{array}$$

and the stability curve of the network is obtained by maximizing this measure over all possible partitions:

$$\begin{array}{c} r\left(t\right)=\max_{H}r\left(t;H\right). \end{array}$$

A good clustering over time *t* will have large stability, with a large trace of *R*_*t*_ over such a time span.

The variation is defined in terms of the asymptotic stability induced by going from the ‘finest’ to the ‘next finest’ partitions is:

$$\begin{array}{c} Variation∼\sum_{i}\sum_{j}\lambda_{2}^{t}\sqrt{d_{i}d_{j}}u_{2i}u_{2j} \end{array}$$

where **u**_2_ is the normalized Fiedler eigenvector with its corresponding eigenvalue *λ*_2_. We refer the mathematical details in deriving these two definitions to [23].

### Hyper-parameters settings for Vicus

There are mainly three hyper-parameters in Vicus: first the number of neighbors *K*, the variance in network construction *σ*, and the diffusion parameter *α*. Details about the meaning of these hyper-parameters can be seen in [30]. In all our experiments, we use the same setting of hyper-parameters as follows:

$$\begin{array}{c} K=10,\;\;\;\sigma =0.5,\;\;\;\alpha =0.9. \end{array}$$

The proposed Vicus is very robust to the choice of *σ* and *α* (S3 Fig). For the choice of *K*, we usually increase *K* as the number of nodes in the networks get larger (S3 Fig). We also provide a range of recommended choices for these hyper-parameters:

$$\begin{array}{c} K\in [5,20],\;\;\;\sigma \in [0.3,0.6],\;\;\;\alpha \in [0.8,0.95] \end{array}$$

We also want to emphasize that, when performing clustering tasks, Vicus does not specify the number of clusters since Vicus is only providing a new form of Laplacian that captures local structures in the network. In our experiments of single-cell applications, we only feed the number of clusters to the clustering algorithms (i.e, K-means algorithm) as the true number of clusters.

## Supporting information

S1 Fig An illustrative example showing Vicus is more robust to noise and outliers compared to Laplacian. Panel A shows the underlying ground-truth network heatmap consisting of 3 connected components. Given this perfect network, we manually add random noise. The random noise is generated from uniform distribution between [0, *δ*]. Larger *δ* indicates bigger magnitude of the noise therefore stronger corruption on the network. Panel B shows an example of the noisy network after corruption when *δ* = 1.2. Panel C is the clustering accuracy measured by NMI if we vary the number of noise strength *δ*.
(EPS)

S2 Fig An illustrative example of comparison between Laplacian and Vicus to illustrate their sensitivity to hyper-parameters used in the construction of similarity network. The first column shows the groundtruth of the data distribution. Panel A is the 3D scattering of the data points used in the experiment. Panel E shows the corresponding 2D ground-truth distribution generating the data. This is also a desired output of low-dimensional embedding we want to recover. Panels B-D shows the results of low-dimensional embedding by Laplacian while Panels F-H are for Vicus using different values of hyper-parameters.
(EPS)

S3 Fig Sensitivity test for three hyper-parameters in Vicus. We apply Vicus on the Buettner data set of single-cell RNA-seq. Panel A shows both NMI and ARI with different choices of number of neighbors *K* with fixed *σ* = 0.5 and *α* = 0.9. Panel B shows both NMI and ARI with different choices of *σ* with fixed *K* = 10 and *α* = 0.9. Panel C shows both NMI and ARI with different choices of *α* with fixed *σ* = 0.5 and *K* = 10.
(TIFF)

S1 File (PDF)
