# Supplementary figures and images for: Vicus: Exploiting local structures to improve network-based analysis of biological data
Source: PLoS Comput Biol. 2017 Oct 12;13(10):e1005621. doi: 10.1371/journal.pcbi.1005621 (PMC5638230; doi:10.1371/journal.pcbi.1005621)

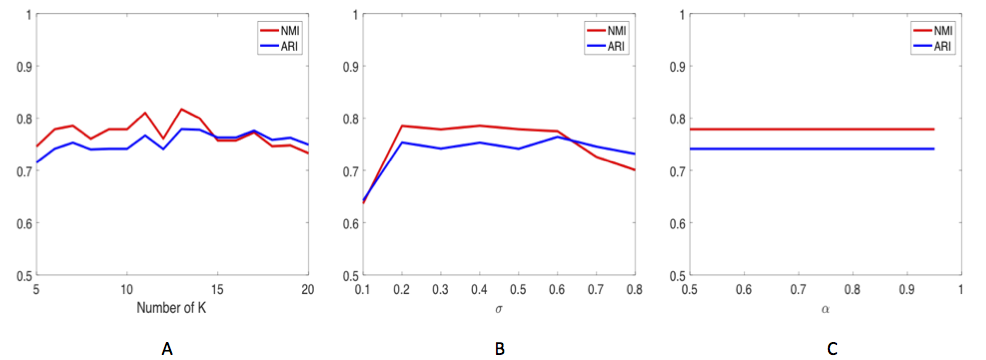

Supplement: S3 Fig — We apply Vicus on the Buettner data set of single-cell RNA-seq. Panel A shows both NMI and ARI with different choices of number of neighbors K with fixed σ = 0.5 and α = 0.9. Panel B shows both NMI and ARI with different choices of σ with fixed K = 10 and α = 0.9. Panel C shows both NMI and ARI with different choices of α with fixed σ = 0.5 and K = 10. (TIFF) [file pcbi.1005621.s003.tiff]
