# Supplementary material for: Vicus: Exploiting local structures to improve network-based analysis of biological data
Source: PLoS Comput Biol. 2017 Oct 12;13(10):e1005621. doi: 10.1371/journal.pcbi.1005621 (PMC5638230; doi:10.1371/journal.pcbi.1005621)
Supplement: S1 File — (PDF) [file pcbi.1005621.s004.pdf]

Vicus: Exploiting Local Structures to Improve Biological Network Analysis

Supplementary Information

Bo Wang[a], Lin Huang[a], Yuke Zhu[a],Anshul Kundaje[a,b], Serafim Batzoglou[a] and Anna Goldenbergc, d

[a] *Department of Computer Science, Stanford University, Stanford, CA, USA*
[b] *Genetics Department, Stanford University, Stanford, CA, USA*
[c] *SickKids Research Institute, Toronto, ON, CA*
[d] *Department of Computer Science, University of Toronto, Toronto, ON, CA*

Supplementary Figures

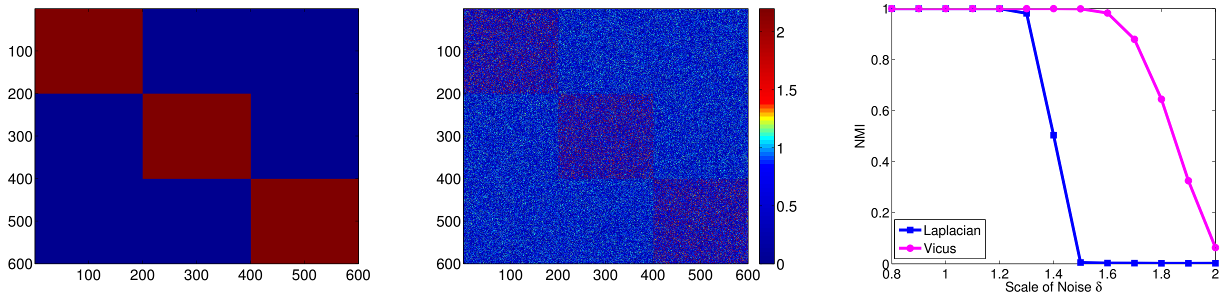

A B C

Figure 1: An illustrative example showing Vicus is more robust to noise and outliers compared to Laplacian. Panel A shows the underlying ground-truth network heatmap consisting of 3 connected components. Given this perfect network, we manually add random noise. The random noise is generated from uniform distribution between $[0, \delta]$. Larger δ indicates bigger magnitude of the noise therefore stronger corruption on the network. Panel B shows an example of the noisy network after corruption when $\delta = 1.2$. Panel C is the clustering accuracy measured by NMI if we vary the number of noise strength δ.

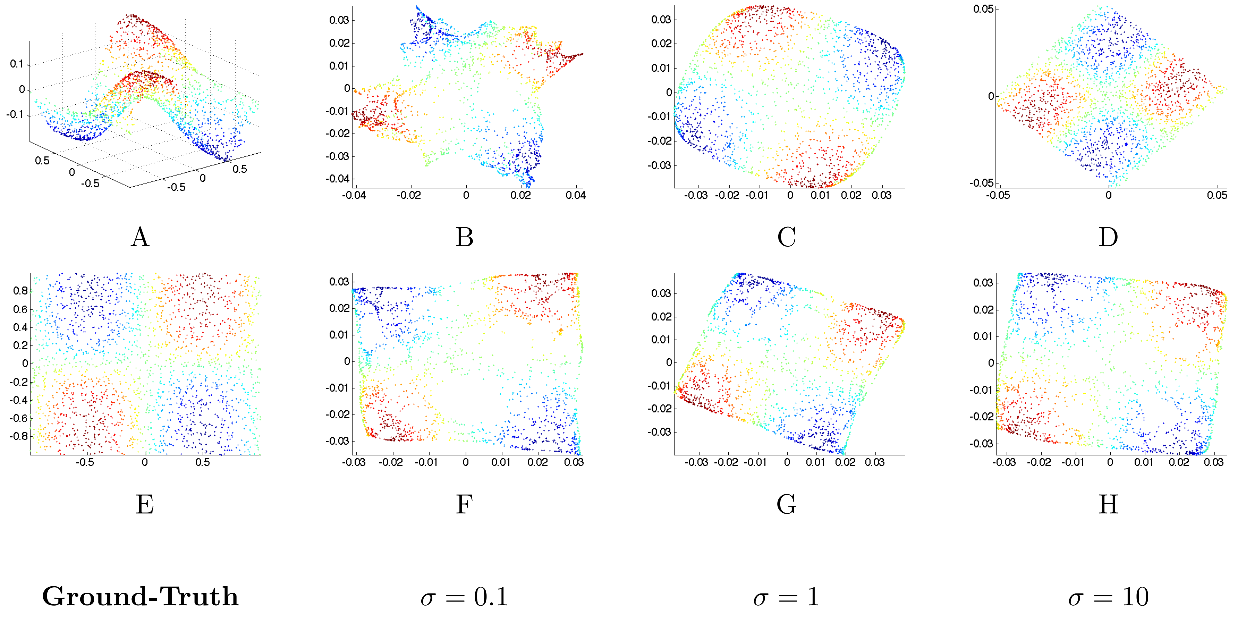

Figure 2: An illustrative example of comparison between Laplacian and Vicus to illustrate their sensitivity to hyper-parameters used in the construction of similarity network. The first column shows the groundtruth of the data distribution. Panel A is the 3D scattering of the data points used in the experiment. Panel E shows the corresponding 2D ground-truth distribution generating the data. This is also a desired output of low-dimensional embedding we want to recover. Panels B-D shows the results of low-dimensional embedding by Laplacian while Panels F-H are for Vicus using different values of hyper-parameters.

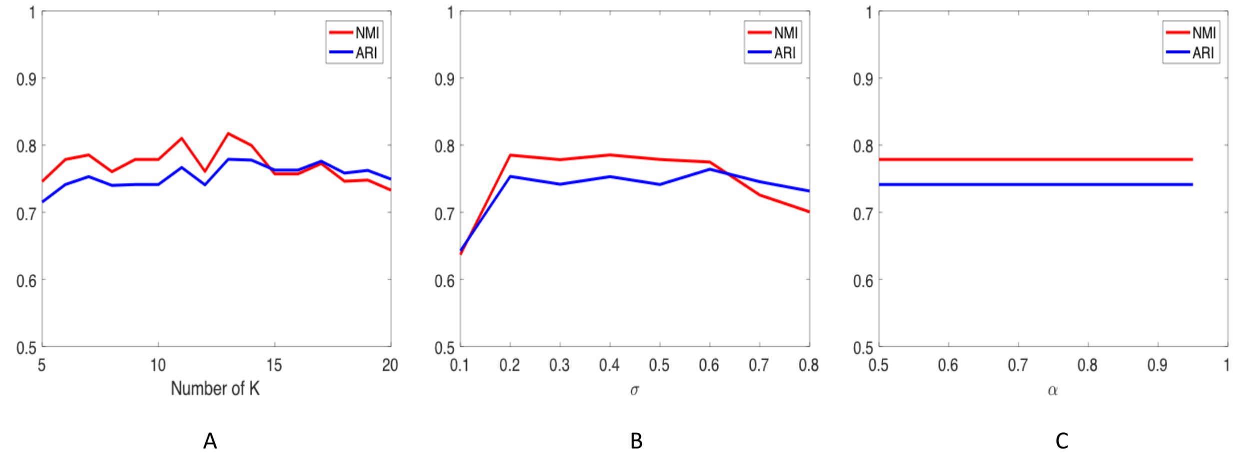

Figure 3: Sensitivity test for three hyper-parameters in Vicus. We apply Vicus on the Buettner data set of single-cell RNA-seq. Panel A shows both NMI and ARI with different choices of number of neighbors K with fixed $\sigma = 0.5$ and $\alpha = 0.9$. Panel B shows both NMI and ARI with different choices of σ with fixed $K = 10$ and $\alpha = 0.9$. Panel C shows both NMI and ARI with different choices of α with fixed $\sigma = 0.5$ and $K = 10$.

Supplementary Note: Implementation Details in Large Scale of Local Spectrum

In this section, we present the details in our implementation of large-scale local spectrum in terms of both memory and speed management.

Memory Issues.

Memory overhead is a serious challenge to the implementations of many spectral methods. Consider a data set with $n \approx 2 \times 10^5$ data points as an example. Storing a full $n \times n$ similarity matrix requires 300GB space. To avoid such a prohibitively significant memory overhead, we implement a memory-efficient version of Local Spectrum in $C/C++$ to demonstrate the feasibility on large data sets and to benchmark the performance.

As the similarity matrix typically has low density, we design a specific data structure to store sparse matrix: a one-dimensional (1D) array linked by two lists. The 1D array contains all the non-zero elements in the matrix, stored in the row-major order; the first list links the first non-zero element in every column; and the second list links the first non-zero element in every row. Let t be the number of non-zero elements in a matrix, the memory overhead of this matrix is $O(t + 2n)$, which is dramatically smaller than the overhead of storing a full matrix $O(n^2)$. Going back to our example, experiments show that the peak memory overhead of applying our memory-efficient implementation is only 5GB.

Based on this data structure, we also implement a series of functions to perform the matrix computations, such as matrix transpose, matrix addition, setting up links to a 1D array. These functions serve as the basic computation blocks in the package; their time complexities are $O(t)$. In the rest of this subsection, we discuss the computational cost of a few steps and omit the straightforward ones.

Computational Time Issues.

We parallelize the KNN identification over all the nodes with OpenMP [1]. For every node x_i in the weighted similarity network we pick the K nearest neighbors of node x_i by maintaining an array containing top K elements while scanning the non-zero elements in the same row as x_i. After we perform this procedure, a sorting is required to store the nearest neighbors in the row-major order. The time complexity is $O(r_i K + K \log K)$, where r_i is the number of non-zero elements in the same row as x_i.

The main computation component of Local Spectrum lies in the calculation of each row of the matrix B in Eq.(4) of the main text. This step can also be parallelized over all the nodes. For every node x_i, let m_i be the number of nodes that belong to the K nearest neighbors of node x_i and the nodes whose K nearest neighbors include x_i. As m_i is usually comparable to K, fitting a few $m_i \times m_i$ two-dimensional arrays into memory is not difficult. Therefore, to achieve high efficiency we create an $(m_i + 1) \times (m_i + 1)$ two-dimensional array that contains the normalized similarities among x_i and its m_i neighbors for the core computation. The array construction costs $O(n + m_i^2)$ time; the sequential core computations are performed on this $2D$ array with time complexity $O(m_i^2)$, except for the matrix inverse. We utilize the Armadillo library version 4.320 (arma.sourceforge.net) to boost the performance of matrix inverse. In terms of Eigen-decomposition and k-means, we reused the source code in the pspectralclustering (Parallel Spectral Clustering [1]) package, which was parallelized using the Message Passing Interface (MPI), to conduct eigen-decomposition and k-means.

[1] www.openmp.org

Time-efficient Version for Mid-Sized Networks

Although many operations in the memory-efficient implementation perform on non-zero elements only, the maintenance of sparse matrices consume considerable running time. Small and medium-size networks, such as Corel, have relatively low memory requirement. For these networks, an efficient way to implement Local Spectrum is to simply keep full matrices in memory. We load the network into a $n \times n$ two-dimensional array. The normalization of this network costs $O(n^2)$ computation time. Sequentially, we identify the K nearest neighbors for every node in the weighted similarity network. The FOR loop over all the nodes is parallelized using OpenMP. For every node x_i, the similarities between x_i and any other nodes are sorted using an insertion sort, while only the K nearest neighbors are kept in the sorted array. Therefore the time complexity is $O(nK)$. Core computation of this version is based on the same data structure as the memory-efficient version, therefore it will not be described here.

Note that, the running time was measured on x 2.67GHz Intel Xeon X5550 processors.

Data used in the experiments We used three data sets whose numbers of samples range from thousands to half million. The first one is called "Corel" which consists of 2074 images of natural objects. It is obtained directly from [1]. It can be seen as an example of Mid-sized network. The second dataset is a common dataset widely used in machine learning, called "MNIST". It contains $80,000$ digit images ranging from "0" to "9". For each The feature is simply the pixel value. All the images are of size 28×28. The last one dataset has more than half million data instances. This dataset describes forest cover types from cartographic variables. Detailed description about this dataset can be found in `https://archive.ics.uci.edu/ml/datasets/Covertype`. Note the last two datasets are downloaded from `http://www.csie.ntu.edu.tw/~cjlin/libsvmtools/datasets/multiclass.html`

Vicus results on large scale networks

One advantage of the global Laplacian is its simplicity. Vicus is more robust and effective but unavoidably suffers from higher computational burden. In this section, we show that, our local spectrum is well suited to distributed and parallel computations and has time complexity comparable to the global Laplacian. We conducted clustering experiments using three benchmark large scale datasets [1] in Table 1. These datasets contain real-world data from various fields. Similarly to [1], our method can be naturally sped up by parallel computing. Detailed theoretical time complexity analysis between Vicus and Laplacian-based spectral clustering is presented in **Materials and Methods**. Our results indicate that local spectrum can achieve higher accuracy of network partitioning with the running time comparable to global spectral clustering (Table 1).

Table 1: A quantitative summarization on the three large scale datasets. Statistics for the three used large scale datasets and results of network clustering by Laplacian and Vicus are reported. Note NMI-L and NMI-V represent the NMI values by Laplacian and Vicus respectively. Similarly, Time-L and Time-V represent the running time by Laplacian and Vicus respectively.

Dataset	#Instances	#Features	#Classes	NMI-L	NMI-V	Time-L	Time-V
Corel	2,074	144	18	0.3836	0.4017	2.0 sec.	2.5 sec.
MNIST	80,000	784	10	0.6510	0.8113	48 sec.	65 sec.
Covtype	581,012	54	7	0.1589	0.2004	7.5 mins	12.9 mins

References

[1] Wen-Yen Chen, Yangqiu Song, Hongjie Bai, Chih-Jen Lin, and Edward Y Chang. Parallel spectral clustering in distributed systems. *Pattern Analysis and Machine Intelligence, IEEE Transactions on*, 33(3):568–586, 2011.
